# Leonurine Regulates Treg/Th17 Balance to Attenuate Rheumatoid Arthritis Through Inhibition of TAZ Expression

**DOI:** 10.3389/fimmu.2020.556526

**Published:** 2020-10-07

**Authors:** Yan-Yi Du, Zhi-Xin Chen, Min-Ying Liu, Qing-Ping Liu, Chang-Song Lin, Cong-Qiu Chu, Qiang Xu

**Affiliations:** ^1^Department of Rheumatology, The First Affiliated Hospital of Guangzhou University of Chinese Medicine, Guangzhou, China; ^2^Guangzhou University of Chinese Medicine, Guangzhou, China; ^3^Chinese Medicine Department, South China Agricultural University Hospital, Guangzhou, China; ^4^Oregon Health & Science University, Portland, OR, United States

**Keywords:** leonurine, Treg Th17, rheumatoid arthritis, TAZ, balance

## Abstract

Leonurine, an active alkaloid extracted from *Herba leonuri*, is reported to have potent anti-inflammatory activity against rheumatoid arthritis (RA). However, the molecular mechanism of action of leonurine in RA remains poorly understood. In this study, we detected 3,425 mRNAs differentially expressed between CD4^+^ T cells of RA patients and those of healthy individuals using microarray raw data mining. Kyoto Encyclopedia of Genes and Genomes enrichment analysis revealed that transcriptional coactivator with PDZ-binding motif (TAZ) regulates a variety of biological processes including T-helper (Th)-17 cell development, and was thus selected for functional verification. In a naïve CD4^+^ T cell differentiation assay, we found that TAZ overexpression was associated with impaired balance between T regulatory (Treg) and Th17 cells *in vitro*. TAZ overexpression increased the levels of the pro-inflammatory cytokines interleukin (IL)-17, IL-1β, and tumor necrosis factor (TNF)-α and decreased that of the anti-inflammatory cytokine IL-10. Leonurine treatment had a direct recovery effect on the impaired balance and reduced the expression of TAZ and led to normalization of IL-17, IL-1β, and TNF-α and IL-10. Furthermore, IL-6 was found to promote the expression of TAZ and receptor activator of nuclear factor kappa-B ligand (RANKL), and RANK. Leonurine significantly inhibited TAZ-mediated expression of RANKL, and RANK and IL-6 in synovial fibroblasts. We conclude that the therapeutic effect of leonurine was through suppression of TAZ led to restoration of Treg/Th17 balance and suppression of synovial fibroblast action.

## Introduction

Rheumatoid arthritis (RA) is the most prevalent systemic autoimmune disease of unknown etiology, with a disability rate of 5 per 1,000 (1). The occurrence and development of the autoimmune response is an extremely complex physiological process involving a variety of immune cells, particularly balance between T regulatory (Treg) and T helper (Th)-17 cells. Previous studies have demonstrated a higher frequency of interleukin (IL)-17-producing Treg cells in the peripheral blood of RA patients compared with that in healthy subjects (2). Van et al. (3) found that CCL21/CCR7 signaling in macrophages promotes joint inflammation and Th17-mediated osteoclast formation in RA. Therefore, the balance between Treg and Th17 cells plays a vital role in the pathogenesis of RA and could be a promising immunomodulatory drug target.

Transcriptional coactivator with PDZ-binding motif (TAZ) is associated with a number of clinical disorders in humans, including cancer and autoimmune diseases (4, 5). Emerging evidence indicates that TAZ regulates the reciprocal differentiation of Treg and Th17 cells, and several studies have found that it plays a critical role in the development of autoimmune diseases (6). Furthermore, there is an intricate interaction between TAZ and inflammatory cytokines. For example, the pro-inflammatory cytokine tumor necrosis factor (TNF)-α/IL-1β can trigger the degradation of YAP/TAZ (7). Furthermore, cell migration and invasion play a vital role in RA (8). Diamantopoulou et al. (9) found that YAP/TAZ promoted the invasion of intestinal epithelial cells.

Leonurine, a unique alkaloid found in the traditional Chinese herb, *Herba leonuri*, exhibits various bioactivities, including anti-apoptotic and anti-inflammatory (10) properties. Jia et al. (11) found that leonurine treatment significantly inhibited the production of the pro-inflammatory cytokines IL-1β, IL-6, and TNF-α in neuroinflammation. Numerous reports have shown its anti-inflammatory function as being vital; leonurine treatment attenuated fibroblast-like synoviocyte-mediated synovial inflammation and joint destruction in RA (12). Although these findings have confirmed that leonurine treatment alleviates RA by inhibiting pro-inflammatory cytokines, the underlying molecular interactions between leonurine and key genes, such as TAZ, have yet to be clarified. The aim of this study was to investigate the potential therapeutic effects of leonurine on RA at the cellular level and elucidate its interactions with TAZ.

## Materials and Methods

### Analysis of Microarray Raw Data Sets From Ncbi Gene Expression Omnibus

Raw data on gene expression (CEL files) were downloaded from the NCBI Gene Expression Omnibus (http://www.ncbi.nlm.nih.gov/geo). The samples (GSE56649_RAW.tar) were divided into two different groups: 13 peripheral blood CD4^+^ T cells of rheumatoid arthritis patients (GSM1366348-GSM1366360) and 9 peripheral blood CD4^+^ T cells of healthy individuals (GSM1366361- GSM1366369). Affymetrix Human Genome U133 Plus 2.0 Array (GPL570) was analyzed using the Affymetrix Transcriptome Analysis Console. Differentially expressed mRNAs were identified considering *p*-value <0.001 and |fold change (FC)| > 1.5. Heatmap and volcano plot were drawn for the differentially expressed mRNAs.

### Kyoto Encyclopedia of Genes and Genomes (KEGG) Pathway Enrichment Analysis

KEGG pathway enrichment analysis of differentially expressed mRNAs was performed using R clusterProfiler (version 3.10.1) from Aipufu.

### Rats and Reagents

Sprague Dawley (SD) rats were obtained from Southern Medical University (Guangzhou, China) (animal production license no. SCXK2016-0041) and housed in a standard pathogen-free environment with free access to food and water. All experiments were carried out according to the National Institutes of Health Guidelines and the Care and Use of Laboratory Animals of Guangzhou University of Chinese Medicine. The following reagents were used: leonurine (Batch number: 0000055070; Sigma), polybrene (Sigma), Lipofectamine 2000 Transfection Reagent (Invitrogen), fetal bovine serum (FBS), Dulbecco's Modified Eagle Medium (DMEM), Opti-MEM, penicillin-streptomycin, phosphate-buffered saline (PBS), and trypsin-EDTA (Gibco), T cell serum-free medium (AdvCell), and special medium for rat synovial cells from Procell (DMEM[H] containing FBS, fibroblast growth additive, penicillin, and streptomycin).

### Primary CD4^+^ T Lymphocyte and Fibroblast-Like Synoviocyte Isolation

Primary CD4^+^ T lymphocytes were isolated from the peripheral blood mononuclear cells of SD rats using Ficoll gradient separation (GE Healthcare, Uppsala, Sweden). T lymphocytes were activated in the presence of plate-bound anti-CD3/anti-CD28 antibodies and cultivated under polarization conditions (13, 14). The purity of the CD4^+^ T lymphocytes was verified using FACS analysis after their isolation and found to always remain above 90% (Supplement Figure 1). Fibroblast-like synoviocytes were isolated by enzymatic digestion of the synovial tissue from the SD rats (15, 16).

### Lentivirus Production and Infection

The 293T packaging cell line was used for lentivirus production. The viruses were collected 48 h after the transfection of the packaging plasmids and the lentivirus-based plasmid using Lipofectamine 2000 (Invitrogen) by following the manufacturer's instructions. After filtering through a 0.45-μM filter, an appropriate volume of virus was used to infect the target cells in the presence of 8 μg/ml polybrene. Subsequently, the target cell lines underwent antibiotic selection dictated by the plasmid. The stable cell lines were selected using puromycin.

### Flow Cytometry

Flow cytometry assays were performed using Becton Dickinson Calibur. Anti-CD4, anti-CD45, anti-Foxp3, anti-IL-17A, anti-CD25, and anti-IgG antibodies were purchased from Abcam (Cambridge, Massachusetts, UK), anti-Foxp3 and anti-ROR-γt were purchased from Invitrogen (Carlsbad, CA, USA). After cell surface staining, cells were fixed and permeabilized and intracellular staining for Foxp3 and RORγt was performed per manufacturer's instruction.

### Pro-inflammatory and Anti-inflammatory Cytokine Assays

IL-17, IL-1β, TNF-α, and IL-10 were quantified using the following enzyme-linked immunosorbent assay (ELISA) kits: Rat IL-1 beta Quantikine ELISA Kit, Rat TNF-α Quantikine ELISA Kit, Rat IL-10 Quantikine ELISA Kit (R&D Systems, Minneapolis, MN, USA), and IL-17 Rat ELISA Kit (Invitrogen, Carlsbad, CA, USA).

### MTT Assays

MTT assays (CellTiter 96 AQueous One Solution Cell Proliferation Assay; Promega, Madison, WI, USA) were used to evaluate cell proliferation, according to the manufacturer's instructions. Cells (1 × 10^4^ cells/well in 100 μL) were seeded in three duplicates in a 96-well plate and cultured for 0, 24, 48, and 72 h. Twenty microliters of Cell Titer 96 AQueous One Solution reagent was added to each well, and the plate incubated for 2 h at 37°C and 5% CO_2_ in the dark. The absorbance was detected at 490 nm using a microplate absorbance reader.

### Quantitative Real-Time PCR (qRT-PCR)

Total RNA was extracted using TRIzol reagent (Invitrogen) and reverse-transcribed to cDNA using a PrimeScript RT Reagent Kit (Takara, Dalian, China), according to the manufacturer's instructions. Real-time PCR was performed using the SYBR Premix ExTaq II Kit (Takara) for 40 cycles on a 7500 Real-Time PCR System (Applied Biosystems, Foster City, CA, USA). The primers for TAZ, receptor activator of nuclear factor kappa-B ligand (RANKL), RANK, and GAPDH (as internal normalization control) were as follows: TAZ, forward 5′-CCACCATCACTTCCACATCG-3′ and reverse 5′- AGAGCAGCTTCCTGCCTCAT-3′; RANKL, forward 5′-CGAGCGCAGATGGATCCTAA-3′ and reverse 5′-GCATTGATGGTGAGGTGTGC-3′; RANK: forward 5′-GCATCCCTTGCAGCTCAACA-3′ and reverse 5′-ATGGAAGAGCTGCAGACCAC-3′; GAPDH, forward 5′-TGGCCGTGGGGCTGCCCAG-3′ and reverse 5′-GGAAGGCCATGCCAGTGAGC-3′. The relative TAZ expression levels were calculated using the 2^Δ*ΔCt*^ method. All PCR experiments were performed in triplicate for each sample, and all experiments were repeated three times.

### Western Blotting

The cells were harvested and lysed using ice-cold lysis buffer (Beyotime, Shanghai, China). The protein concentration was then determined using a protein assay kit (Keygentec, Nanjing, China). The denatured proteins (20 μg) were separated using sodium dodecyl sulfate-polyacrylamide gel electrophoresis (SDS-PAGE) and transferred onto polyvinylidene fluoride membranes (Millipore, Billerica, MA, USA). After blocking, the membranes were incubated at 4°C overnight with anti-TAZ antibody, anti-RANKL antibody, anti-RANK antibody, and anti-GAPDH antibody (Abcam, Cambridge, MA, USA). Next, the membranes were incubated with secondary antibodies for 2 h at 25°C. The bound proteins were visualized by enhanced chemiluminescence (Thermo Scientific) and detected using an imaging system (DNR Bio-Imaging Systems Ltd., Jerusalem, Israel), with GAPDH as the loading control.

### *In vitro* Migration and Invasion Assays

Cell migration and invasion assays were carried out using the BD 24-well Transwell chamber (BD Biosciences, San Jose, CA, USA). The cells (1 × 10^5^) were harvested and placed in serum-free medium in the top chamber coated without or with Matrigel (BD Biosciences, San Jose, CA, USA). Complete medium supplemented with 10% FBS was added to the bottom chamber. After incubating for 48 h at 37°C and 5% CO_2_, the cells that migrated to and invaded the reverse side of the chamber inserts were fixed with methanol for 30 min and then stained with crystal violet for 15 min. The number of cells was measured in three randomly selected high-power fields across the center and periphery of the membrane under a light microscope (Olympus, Tokyo, Japan). All experiments were performed in triplicate.

### Statistical Analysis

All data are expressed as the mean ± standard deviation (SD) and analyzed using SPSS version 19.0 (SPSS Inc., Chicago, IL, USA). For independent two-group analyses, the Student's *t*-test was used.

### Data Availability

The microarray data [GSE56649] for this study can be found in a public repository on the GEO website (http://www.ncbi.nlm.nih.gov/geo). The authors declare that all the other data supporting the findings of this study are available within the article and its Supplementary Information files and from the corresponding authors on reasonable request.

## Results

### TAZ Is Highly Expressed in CD4^+^ T Cells of RA Patients

**Figure 1:**
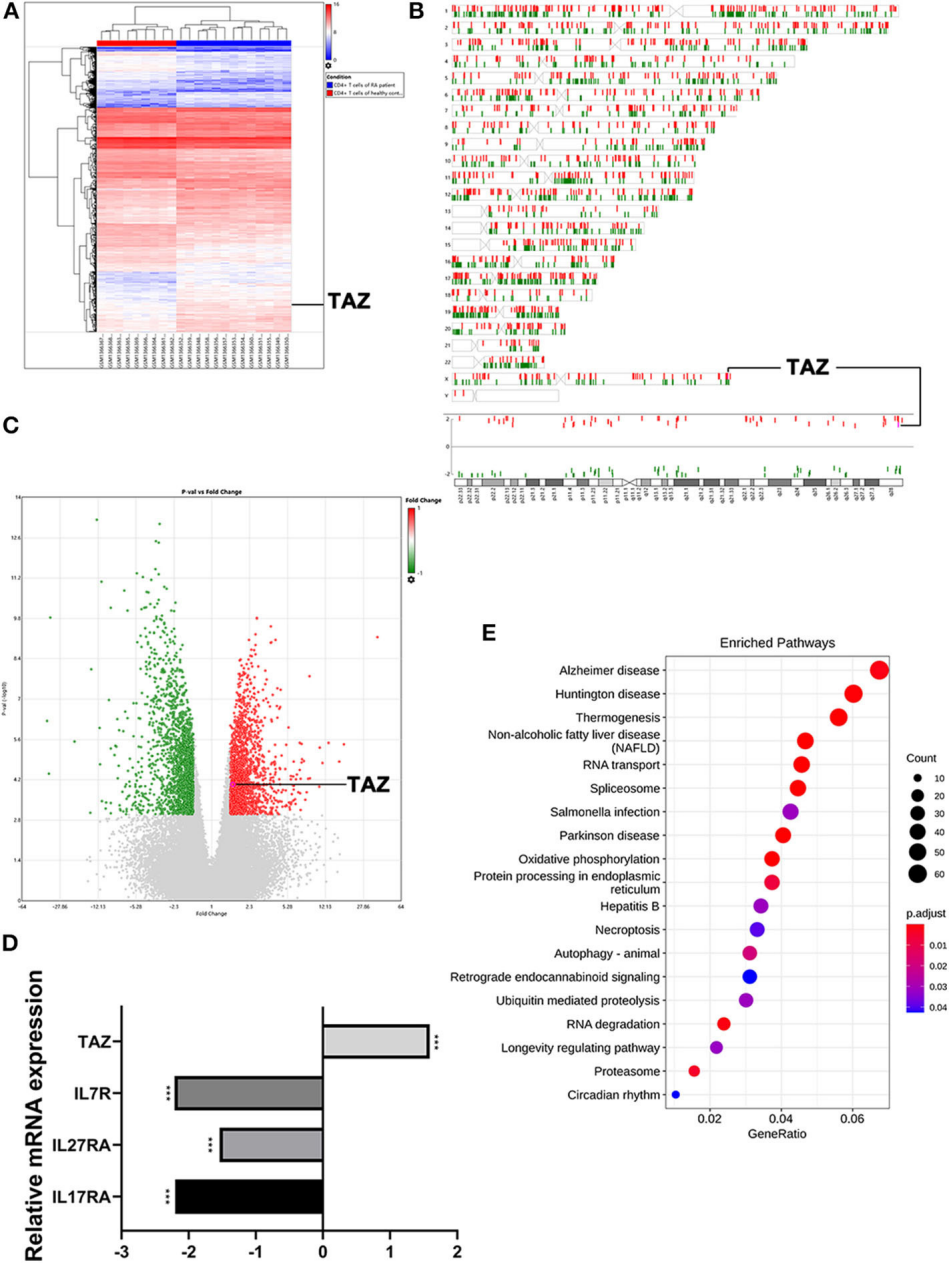
Highly expressed TAZ screened in CD4^+^ T cells of rheumatoid arthritis patients using microarray raw data analysis. **(A)** Heatmap of the hierarchical cluster analysis of differentially expressed miRNAs between 13 CD4^+^ T cells of rheumatoid arthritis patients and 9 CD4^+^ T cells of healthy individuals using *P* < 0.001 and |FC| > 1.5 as the cutoff (bright blue, under-expression; white, no change; bright red, over-expression). **(B,C)** Chromosome summary plot and volcano plot of the microarray data. **(D)** The expression of TAZ was higher in CD4^+^ T cells of rheumatoid arthritis patients. **(E)** Top 20 statistics of KEGG pathway enrichment analysis. ***P < 0.001.

Three thousand four hundred and twenty-five mRNAs were found to be differentially expressed between the CD4^+^ T cells of RA patients (*n* = 13) and healthy individuals (*n* = 9) using hierarchical clustering analysis (Figure 1A) followed by construction of a chromosome summary plot (Figure 1B) and a volcano plot (Figure 1C). TAZ expression was up-regulated in the CD4^+^ T cells of RA patients, whereas the expression of IL-7R, IL-27RA, and IL-17RA, which are involved in innate defense mechanisms, was reduced (Figure 1D). Moreover, the KEGG pathway enrichment analysis showed that the mRNAs were enriched in various disease pathways (Figure 1E). Previous studies have demonstrated that TAZ acts as an activator of the Hippo signaling pathway in various diseases (4, 5). Based on these findings, TAZ was selected for subsequent experiments.

### High Purity of Isolated T Cells Determined by Flow Cytometry Analysis of Surface CD4 or CD45 Staining

The purity of the isolated T cells was found to be >90% by flow cytometry analysis of surface CD4 or CD45 staining (Supplement Figure 1).

### Leonurine Treatment Reduces TAZ Expression in T Cells

**Figure 2:**
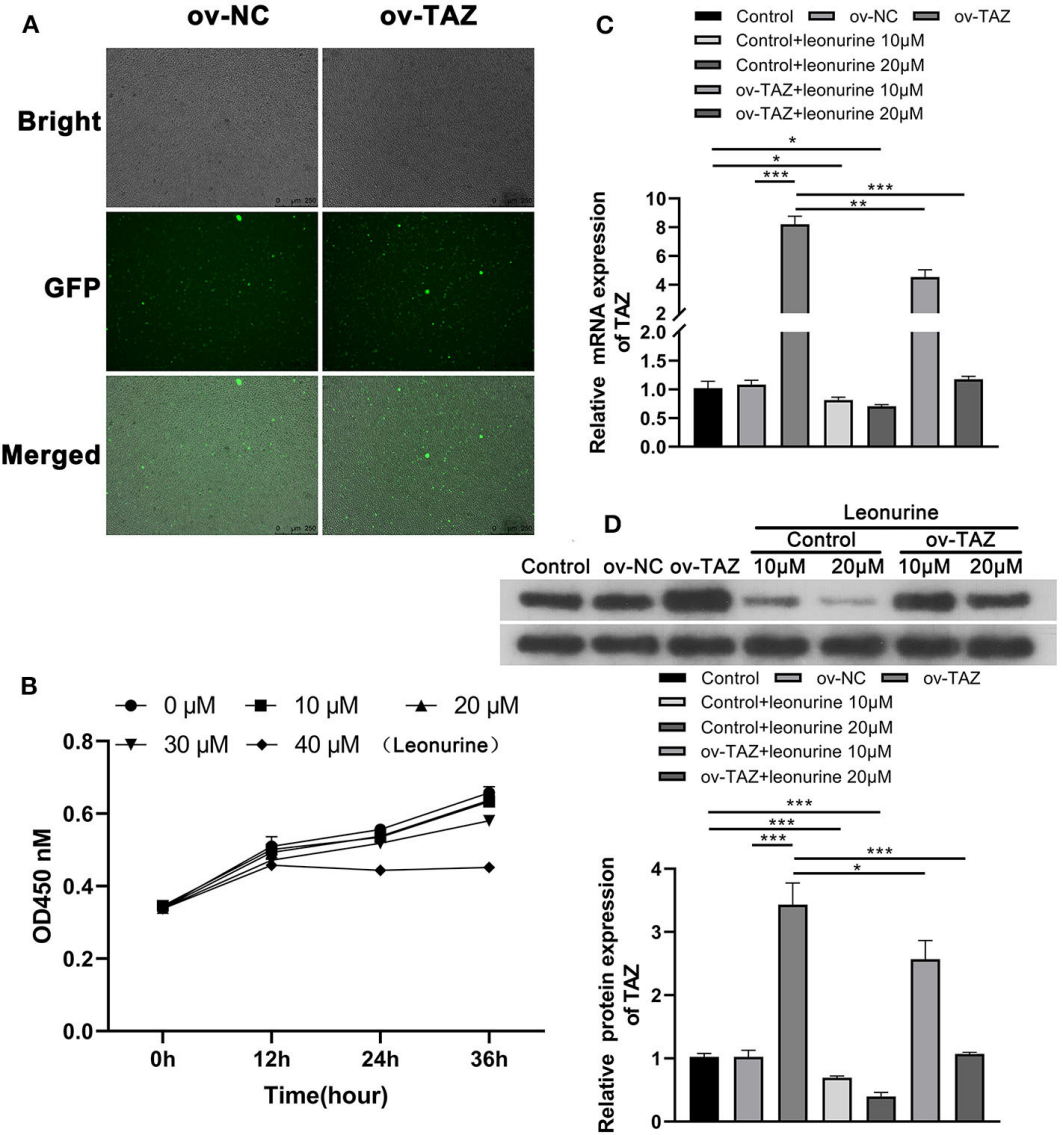
Leonurine treatment reduced the expression of TAZ in T cells. **(A)** 293 T-cells stably expressing TAZ previously established by transfection were used as a positive control (ov-TAZ) and a negative control (ov-NC) (magnification, × 100). **(B)** MTT assays showed that leonurine had the least cytotoxicity at 20 μM. There was no difference in suppression of proliferation between the leonurine 20 and 10 μM groups (*P* > 0.05), while leonurine 30 μM group was significantly different from the leonurine 20 μM group (*P* < 0.05), and the leonurine 0 μM groupwas used as a negative control. **(C,D)** qPCR and Western blot results showed that the expression of TAZ in the ov-TAZ group was higher than that in the ov-NC group. Leonurine treatment reduced the expression of TAZ. Furthermore, leonurine was able to suppress TAZ expression in TAZ-overexpressed T cells. Results are expressed as average of 3 experiments. *P < 0.05, **P < 0.01, and ***P < 0.001.

To determine the role of TAZ in T cells, two sets of T cell lines were analyzed: T cells from the peripheral blood of SD rats stably over-expressing TAZ (ov-TAZ) and a negative control using lentivirus (ov-NC) (Figure 2A). The optimal concentration of leonurine was found to be 20 μM based on MTT assay results (Figure 2B). The efficiency of TAZ overexpression in the stable cell lines was confirmed by qPCR and Western blot analyses, which showed that leonurine treatment reduced the expression of TAZ in T cells (Figures 2C,D).

### Leonurine Treatment Reverses Treg/Th17 Imbalance Induced by TAZ

**Figure 3:**
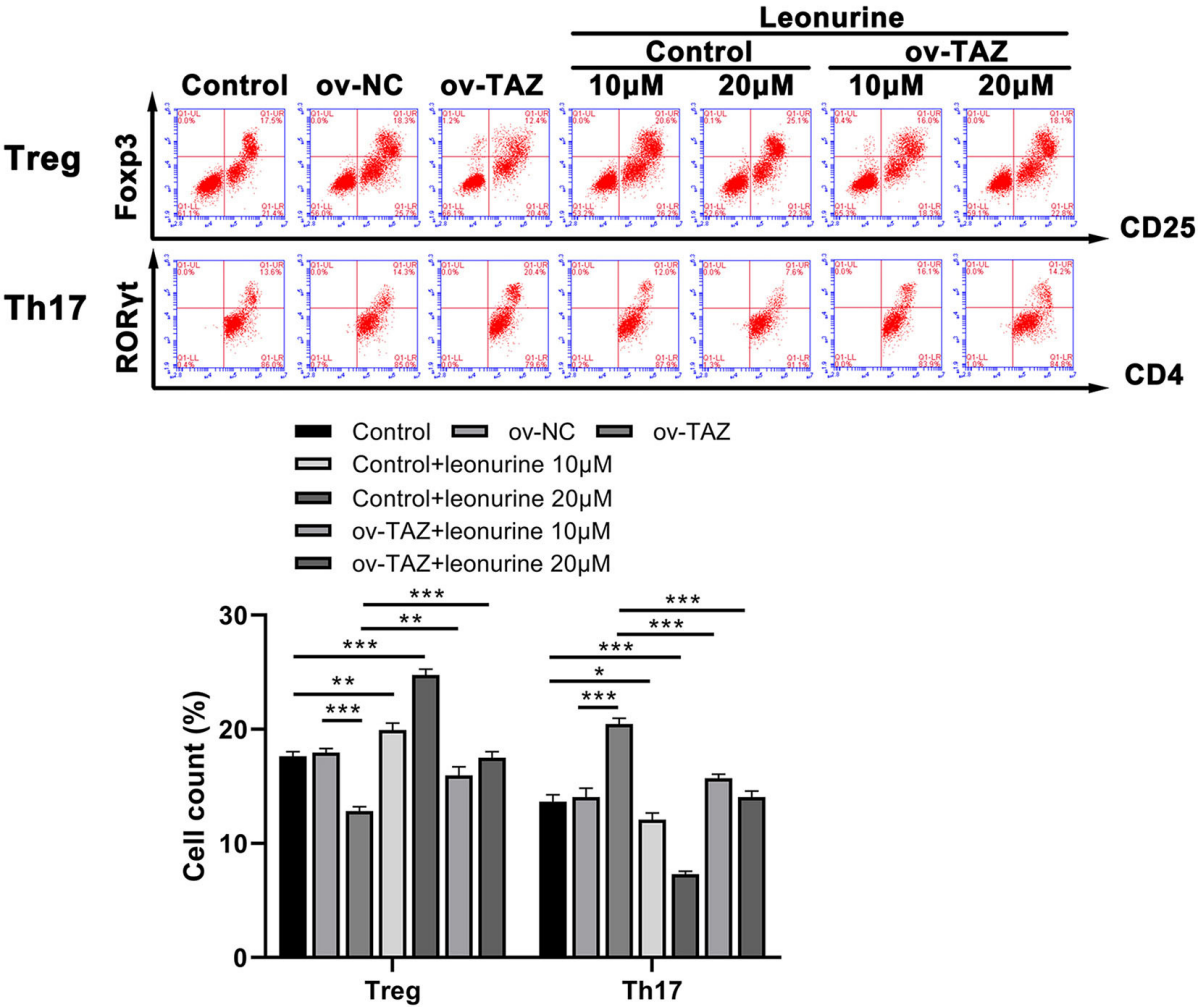
Flow cytometry results showed that the frequency of Treg cells (expressing Foxp3) in the ov-TAZ group was lower than that in the ov-NC group, whereas the frequency of Th17 cells (RORγt expressing) in the ov-TAZ group was higher than that in the ov-NC group. When leonurine was added, it increased the Treg cell frequency and decreased that of Th17 cells. Furthermore, leonurine increased Treg cell frequency in the ov-TAZ group and decreased that of Th17 cells. Results are expressed as average of 3 experiments. *P < 0.05, **P < 0.01, and ***P < 0.001.

To investigate the influence of TAZ on the Treg/Th17 balance, we evaluated the frequency of CD4^+^ RORγt^+^ cells and CD25^+^ Foxp3^+^ cells in naïve T cells obtained from the peripheral blood of SD rats. The frequency of Th17 cells in the ov-TAZ group was higher than that in the ov-NC group, whereas that of Treg cells was lower. These results indicate that TAZ induced a Treg/Th17 imbalance. However, leonurine treatment reduced the TAZ-mediated Treg/Th17 balance. When added directly to T cells, leonurine also increased the proportion of Treg cells and reduced that of Th17 cells (Figure 3).

### Leonurine Treatment Reduces Inflammation Induced by TAZ

**Figure 4:**
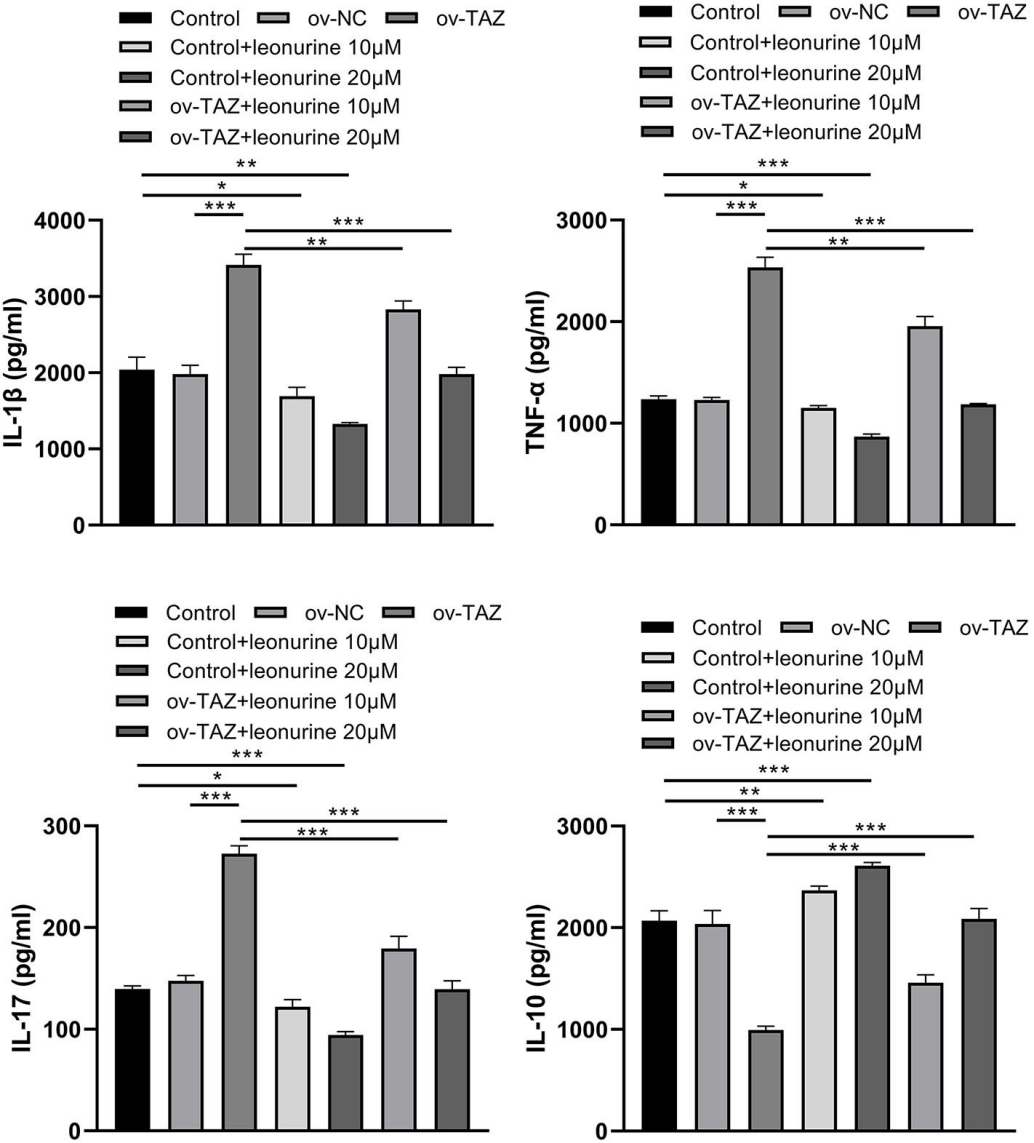
ELISA assay showing TAZ-mediated inflammatory cytokine production by CD4^+^ T cells and suppression by leonurine. Compared with those in the ov-NC group or NC group, the protein levels of the pro-inflammatory cytokines IL-17, IL-1β, and TNF-α in the ov-TAZ group increased, whereas those of the anti-inflammatory cytokine IL-10 decreased. After leonurine treatment, the protein levels of IL-17, IL-1β, and TNF-α in the control group and ov-TAZ group decreased, whereas that of IL-10 increased. Results are expressed as average of 3 experiments. *P < 0.05, **P < 0.01, and ***P < 0.001.

We assessed the protein levels of major pro- and anti-inflammatory cytokines to determine whether TAZ causes inflammation and whether leonurine has an anti-inflammatory effect. The direct addition of leonurine was found to down-regulate T cell inflammation. Compared with that in the ov-NC group, the expression of IL-17, IL-1β, and TNF-α in the ov-TAZ group was found to increase, whereas the level of IL-10 decreased. Thus, leonurine appears to have a significant therapeutic effect on the inflammation caused by TAZ overexpression (Figure 4).

### Leonurine Treatment Diminishes Inflammation in Arthritis Caused by TAZ Overexpression in Synovial Cells

**Figure 5:**
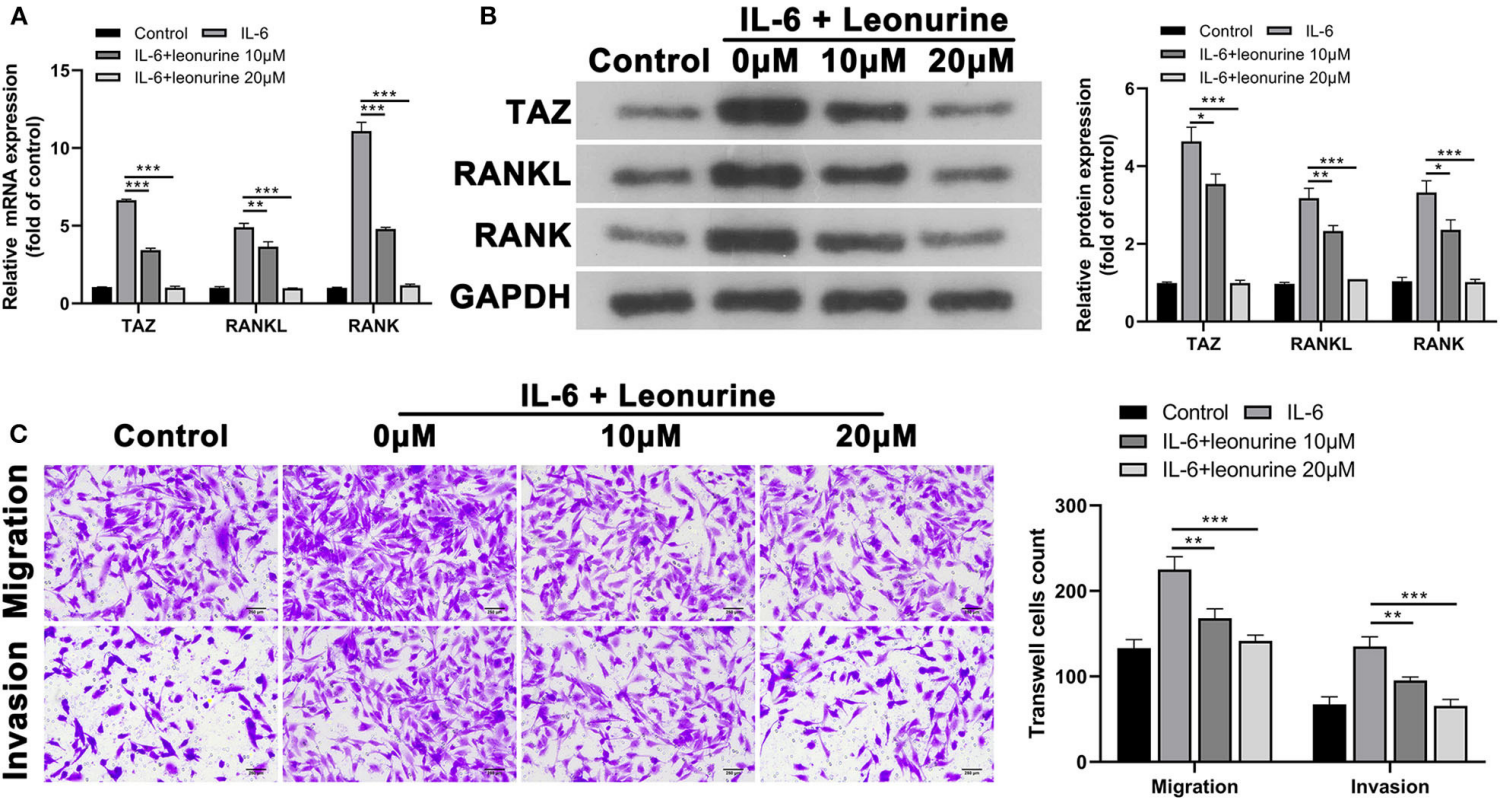
Suppressive effect of leonurine on TAZ-mediated migration and invasion of synovial fibroblast-like cells. **(A,B)** qPCR and Western blot analysis showed that compared with that in the control group, TAZ, RANKL, and RANK by synovial fibroblast-like cells in the IL-6 group were all up-regulated. However, leonurine treatment (10 and 20 μM) reduced the effects of IL-6 on TAZ/RANKL/RANK in a dose dependent manner. **(C)** Transwell (magnification, × 200) analysis showed that IL-6 promoted the migration and invasion ability of fibroblast-like cells, whereas leonurine treatment reduced the effect of IL-6. Results are expressed as the average of 3 experiments. *P < 0.05, **P < 0.01, and ***P < 0.001.

Based on the MTT analysis results, leonurine doses of 10 and 20 μM were selected for subsequent expression verification and to determine the effect of leonurine on synovial fibroblast-like cells. To evaluate the role of TAZ in RA, we investigated the expression of TAZ, RANKL, and RANK in synovial fibroblast-like cells (17, 18). The results of the qPCR and Western blot analysis indicated that IL-6 promotes the expression of TAZ, RANKL, and RANK compared with that in the control group (Figures 5A,B). The administration of leonurine reversed these effects of L-6 on synovial fibroblasts. Leonurine diminished IL-6-induced TAZ expression and subsequently RANK and RANKL expression. Furthermore, transwell analysis also confirmed that IL-6 promoted migration and invasion of synovial fibroblast-like cells were reversed by leonurine (Figure 5C).

## Discussion

RA is a chronic systemic immune-mediated inflammatory disorder with a prevalence of ~1%, ranking among the top 15% diseases that cause disabilities worldwide (19). Leonurine, a plant-derived compound, has been investigated previously with the aim to develop a drug able to reduce inflammation while not significantly suppressing the immune response to reduce its side effects for the treatment of RA. We have shown that leonurine attenuates RA inflammation (12), but the molecular mechanism of action of leonurine is not fully elucidated. In this study, we demonstrated that the anti-inflammatory effect of leonurine is through its suppression of TAZ, a molecule involves Th17 development and imbalance between Treg and Th17 cells. To the best of our knowledge, this study is the first to report that leonurine treatment regulates the Treg/Th17 balance to alleviate RA by inhibiting TAZ expression.

Two types of key immune cells, Treg and Th17 cells, regulate the initiation and development of RA. Li et al. (20) found that arsenic trioxide improves the Treg/Th17 balance by modulating STAT3 in treatment-naïve RA patients. Previous studies have demonstrated that CCL21/CCR7 signaling in macrophages promotes joint inflammation and Th17-mediated osteoclast formation in RA (3). In the present study, the Treg/Th17 imbalance was confirmed to exacerbate the inflammatory responses in RA. The protein levels of major pro- and anti-inflammatory cytokines were assessed as key indicators of the degree of inflammation after induction of Treg/Th17 imbalance. In the context of Treg/Th17 imbalance, the levels of IL-17, IL-1β, and TNF-α in the ov-TAZ group were found to be increased, whereas the expression of IL-10 was reduced. Thus, given recent advances in IL-17/IL-1β/TNF-α/IL-10-based therapeutic drugs, Treg and Th17 cells show promise as immunomodulatory drug targets for the treatment of RA.

TAZ acts as an oncogenic protein by promoting cell proliferation and migration. TAZ is also directly related to the differentiation of Treg/Th17 cells (6). In this study, TAZ was confirmed to induce a Treg/Th17 imbalance in patients with RA by promoting Th17 cell differentiation and inhibiting Treg cell differentiation. However, both of these effects of TAZ were reversed after treatment with leonurine.

Taken together, this study contributes to improvement of our understanding of the role of TAZ in RA. The therapeutic effect of leonurine in RA is via down-regulation of TAZ. Further studies are required to delineate how leonurine inhibits TAZ expression. Understanding of the fine regulation pathway of TAZ will provide potential therapeutic target points to block TAZ gene expression.

## Data Availability Statement

The datasets presented in this study can be found in online repositories. The names of the repository/repositories and accession number(s) can be found in the article/Supplementary Material.

## Ethics Statement

The animal study was reviewed and approved by Experimental animal ethics committee of Guangzhou University of traditional Chinese Medicine. Written informed consent was obtained from the owners for the participation of their animals in this study.

## Author Contributions

QX, C-QC, and C-SL conceived the manuscript. QX, Y-YD, and Z-XC drafted the manuscript. Y-YD and Z-XC did the experiment of the study. M-YL and Q-PL did part of the experimental work. QX and C-QC revised the manuscript.

## Conflict of Interest

The authors declare that the research was conducted in the absence of any commercial or financial relationships that could be construed as a potential conflict of interest.

## Supplementary Material

Supplement Figure 1 The purity of the isolated CD4^+^ T cells.
