# Supplementary figures and images for: Leonurine Regulates Treg/Th17 Balance to Attenuate Rheumatoid Arthritis Through Inhibition of TAZ Expression
Source: Front Immunol. 2020 Oct 7;11:556526. doi: 10.3389/fimmu.2020.556526 (PMC7575723; doi:10.3389/fimmu.2020.556526)

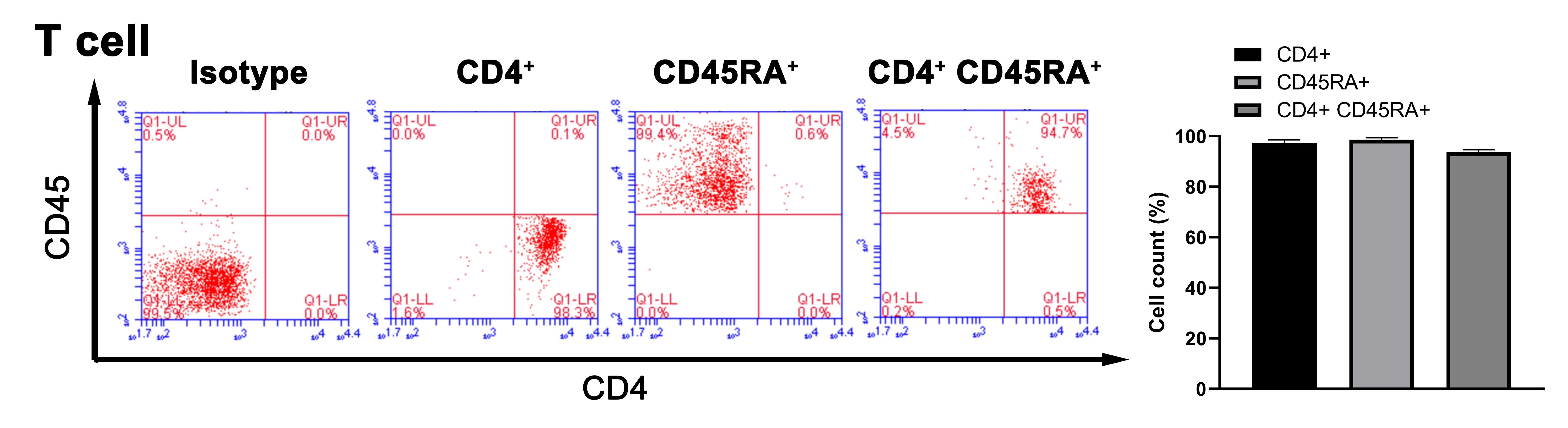

Supplement: Supplement Figure 1 — The purity of the isolated CD4+ T cells. [file Image_1.TIF]
